# Investigating intestinal mast cell dynamics during acute heat stress in growing pigs

**DOI:** 10.1093/jas/skae030

**Published:** 2024-01-30

**Authors:** Edith J Mayorga, Sonia Rodriguez-Jimenez, Megan A Abeyta, Brady M Goetz, Julie Opgenorth, Adam J Moeser, Lance H Baumgard

**Affiliations:** Department of Animal Science, Iowa State University, Ames, IA 50011, USA; Department of Animal Science, Iowa State University, Ames, IA 50011, USA; Department of Animal Science, Iowa State University, Ames, IA 50011, USA; Department of Animal Science, Iowa State University, Ames, IA 50011, USA; Department of Animal Science, Iowa State University, Ames, IA 50011, USA; Department of Large Animal Clinical Sciences, College of Veterinary Medicine, Michigan State University, East Lansing, MI 48824, USA; Department of Animal Science, Iowa State University, Ames, IA 50011, USA

**Keywords:** corticotropin-releasing factor, cortisol, histamine, inflammation, stress

## Abstract

Objectives were to examine the temporal pattern of intestinal mast cell dynamics and the effects of a mast cell stabilizer (ketotifen [**Ket]**) during acute heat stress (**HS**) in growing pigs. Crossbred barrows (*n* = 42; 32.3 ± 1.9 kg body weight [**BW**]) were randomly assigned to 1 of 7 environmental-therapeutic treatments: (1) thermoneutral (**TN**) control (**TNCon**; *n* = 6), (2) 2 h HS control (**2 h HSCon**; *n* = 6), (3) 2 h HS + Ket (**2 h HSKet**; *n* = 6); (4) 6 h HSCon (*n* = 6), (5) 6 h HSKet (*n* = 6), (6) 12 h HSCon (*n* = 6), or (7) 12 h HSKet (*n* = 6). Following 5 d of acclimation to individual pens, pigs were enrolled in two experimental periods (**P**). During P1 (3 d), pigs were housed in TN conditions (21.5 ± 0.8 °C) for the collection of baseline measurements. During P2, TNCon pigs remained in TN conditions for 12 h, while HS pigs were exposed to constant HS (38.1 ± 0.2 °C) for either 2, 6, or 12 h. Pigs were euthanized at the end of P2, and blood and tissue samples were collected. Regardless of time or therapeutic treatment, pigs exposed to HS had increased rectal temperature, skin temperature, and respiration rate compared to their TNCon counterparts (1.9 °C, 6.9° C, and 119 breaths/min; *P* < 0.01). As expected, feed intake and BW gain markedly decreased in HS pigs relative to their TNCon counterparts (*P* < 0.01). Irrespective of therapeutic treatment, circulating corticotropin-releasing factor decreased from 2 to 12 h of HS relative to TNCon pigs (*P* < 0.01). Blood cortisol increased at 2 h of HS (2-fold; *P* = 0.04) and returned to baseline by 6 h. Plasma histamine (a proxy of mast cell activation) remained similar across thermal treatments and was not affected by Ket administration (*P* > 0.54). Independent of Ket or time, HS increased mast cell numbers in the jejunum (94%; *P* < 0.01); however, no effects of HS on mast cell numbers were detected in the ileum or colon. Jejunum and ileum myeloperoxidase area remained similar among treatments (*P* > 0.58) but it tended to increase (12%; *P* = 0.08) in the colon in HSCon relative to TNCon pigs. Circulating lymphocytes and basophils decreased in HSKet relative to TN and HSCon pigs (*P* ≤ 0.06). Blood monocytes and eosinophils were reduced in HS pigs relative to their TNCon counterparts (*P* < 0.01). In summary, HS increased jejunum mast cell numbers and altered leukocyte dynamics and proinflammatory biomarkers. However, Ket administration had no effects on mast cell dynamics measured herein.

## Teaser

Mast cell activation has been implicated in the pathogenesis of various stress-related intestinal inflammatory disorders. This study sheds light on a plausible link between heat stress (HS) and intestinal mast cell infiltration, suggesting mast cells may have a central role in the etiology of intestinal barrier dysfunction during HS.

## Introduction

Environmental stressors and heat stress (**HS**), in particular, impose a huge economic burden on the animal agriculture sector (St-Pierre et al., 2003; Key and Sneeringer, 2014). These pecuniary losses are mainly associated with reduced growth rates, decreased milk yield, altered carcass characteristics, decreased reproduction, increased mortality/morbidity, and reduced facility efficiency (Baumgard and Rhoads, 2013; Ross et al., 2015). The exact etiological mechanisms underlying reduced animal productivity and health during HS are not clear; however, a growing body of evidence suggests that intestinal barrier dysfunction and the ensuing immune activation are the main contributors (Pearce et al., 2013; Koch et al., 2019; Nanto-Hara et al., 2020). During HS, decreased intestinal barrier integrity has been traditionally associated with a circulatory event where animals maximize heat dissipation by redistributing blood flow toward the periphery and away from splanchnic tissues (i.e., gastrointestinal tract and liver; Hall et al., 1999). Consequently, intestinal hypoperfusion and concomitant hypoxia ostensibly cause oxidative stress, enterocyte damage, and tight junction disruption (Hall et al., 2001; Lambert et al., 2002).

In addition to intestinal hypoperfusion, it is becoming increasingly clear that generalized stress might also alter intestinal epithelial integrity. Stress activates the hypothalamic-pituitary-adrenal (**HPA**) axis, stimulating the release of central and peripheral stress mediators, including corticotropin-releasing factor (**CRF**; Charmandari et al., 2005). The CRF system is associated with various stress-related gastrointestinal disorders, including gastrointestinal dysmotility, increased mucosal permeability, and intestinal inflammation (Santos et al., 2001; Wallon et al., 2008; Overman et al., 2012). This pathophysiology appears to be largely regulated by mast cells (Wouters et al., 2016). Mast cells are important mediators of innate and adaptive immunity, and leukocytes within each branch express CRF receptors, which may explain the association between psychological anxiety and intestinal distress (Smith et al., 2010; Ayyadurai et al., 2017). Upon CRF stimulation, mast cells synthesize and release various mediators, including histamine, proteases (e.g., chymase and tryptase), and proinflammatory cytokines that negatively compromise intestinal barrier function (as reviewed by Albert-Bayo et al., 2019).

Interestingly, CRF activation, mast cell degranulation, and the ensuing intestinal barrier dysfunction have been implicated in various seemingly unrelated situations, including weaning (Moeser et al., 2007a, b; Pohl et al., 2017), cold (Saunders et al., 2002), and social stress (Li et al., 2017). Further, preventing stress-induced mast cell activation by administering mast cell stabilizers has been shown to reduce gut permeability and improve pig growth (Moeser et al., 2007b; Mereu et al., 2015). While evidence supporting mast cell activation as a mediator of gut barrier dysfunction during HS is scarce, the common intestinal pathology to both HS and physiological/emotional stress suggests neuroendocrine mechanisms play an essential role in compromising intestinal barrier function during a heat load. Therefore, we hypothesized that pigs exposed to acute HS will have increased biomarkers of mast cell activation, and administering a mast cell stabilizer would minimize mast cell degranulation and reduce the inflammatory response to HS. Thus, the study objectives were to investigate the temporal pattern of mast cell dynamics and evaluate the therapeutic effects of mast cell stabilization on metabolism and inflammatory biomarkers during acute HS in growing pigs.

## Materials and Methods

All experimental procedures followed the guidelines for the ethical and humane use of animals for research according to the Guide for the Care and Use of Agricultural Animals in Research and Teaching (FASS, 2010) and were approved by the Iowa State University Institutional Animal Care and Use Committee (#20-140).

### Animals, housing, and experimental design

Forty-two crossbred barrows (32.3 ± 1.9 kg body weight [**BW**]) were utilized in the current study conducted at the Iowa State University Swine Nutrition Farm facility (Ames, IA). Based on BW, pigs were randomly assigned to 1 of 7 environmental-therapeutic treatments: (1) thermoneutral (**TN**) control (**TNCon**; *n* = 6), (2) 2 h HS control (**2 h HSCon**; *n *= 6), (3) 2 h HS + Ketotifen (**Ket**; **2 h HSKet**; *n* = 6), (4) 6 h HSCon (*n* = 6), (5) 6 h HSKet (*n* = 6), (6) 12 h HSCon (*n* = 6), or (7) 12 h HSKet (*n* = 6). Pigs were allocated to one of two environmentally controlled rooms and housed in individual pens (57 × 221 cm) equipped with a stainless-steel feeder and a nipple drinker. Pigs were fed a standard diet formulated to meet or exceed the requirements for growing pigs for essential amino acids, minerals, and vitamins (NRC, 2012; Table 1). Feed and water were provided ad libitum during the entire experiment.

**Table 1.** Ingredient composition of diet (as-fed basis)

Ingredient	Percentage
Corn	72.78
Soybean meal (47.5% CP)	23.20
Soybean oil	0.80
Limestone	0.92
Monocalcium phosphate	1.12
l-Lys HCl	0.30
dl-Met	0.06
l-Thr	0.07
NaCl	0.40
Vitamin premix^1^	0.20
Mineral premix^2^	0.15

^1^Vitamin premix provided the following per kilogram of complete diet: 6,125 IU vitamin A, 700 IU vitamin D3, 50 IU vitamin E, 3 mg vitamin K, 11 mg riboflavin, 56 mg niacin, 27 mg pantothenic acid, 24 mg vitamin B12.

^2^Mineral premix provided the following per kilogram of complete diet: 165 mg Fe (ferrous sulfate), 165 mg Zn (zinc sulfate), 39 mg Mn (manganese sulfate), 16.5 mg Cu (copper sulfate), 0.3 mg I (calcium iodate), 0.3 mg Se (sodium selenite).

Following 5 d of acclimation to individual pens, pigs were enrolled in two experimental periods (**P**). During P1 (3 d), pigs were housed in TN conditions (21.5 ± 0.8 °C; 39.5 ± 6.4% relative humidity) for the collection of baseline body temperature indices and production parameters. During P2, HS pigs were exposed to either 2, 6, or 12 h of constant HS (38.1 ± 0.2 °C; 22.4 ± 2.2% relative humidity; 82.4 ± 0.6 temperature-humidity index), while TNCon pigs remained in TN conditions for 12 h. During both P1 and P2, room temperature and humidity were monitored and recorded every 5 min by a data logger (Lascar EL-USB-2LCD, Erie, PA).

### Therapeutic treatments

Treatments included an intramuscular injection of sterile saline (**Con**; 2.5 mL) or the mast cell stabilizer drug Ket (10 mg/kg BW; Ketotifen Fumarate; Cayman Chemical, Ann Arbor, MI). Ket was dissolved with absolute ethanol and then diluted with sterile water to reach a final concentration of 370 mg/mL; the total volume of Ket administered per pig was approximately 1.5 mL. Pigs received their respective Con or Ket treatment approximately 1 h before the initiation of the environmental challenge. Using a similar mast cell stabilizer, a comparable dose regimen reduced intestinal mast cell numbers and ameliorated intestinal barrier dysfunction in response to weaning stress in pigs (Moeser et al., 2007b; Mereu et al., 2015).

### Body temperature indices

Rectal temperature (**T**_**R**_), skin temperature (**T**_**S**_), and respiration rate (**RR**) were obtained twice daily (~0700 and 1700 hours) during P1 and at 0, 2, 6, and 12 h during P2. Rectal temperature was measured using a digital thermometer (digital thermometer with flex tip, accuracy: ±0.1 °C; Jorgensen Labs Inc., Loveland, CO). Skin temperature was measured at the rump area using an infrared thermometer (Southwire digital thermometer, accuracy: ±2 °C; Southwire Company, LLC., Carrollton, GA), and RR was determined by counting flank movements for 15 s and multiplied by 4 to obtain breaths per minute (**bpm**).

### Production parameters

Feed intake (**FI**) was measured daily during P1 and at 2, 6, and 12 h during P2 as feed disappearance. BWs were recorded at the beginning and the end of the acclimation period, at the beginning of P2, and immediately before euthanasia. BW change was calculated by subtracting final BW from the BW recorded before the initiation of the environmental challenge.

### Blood sampling and analysis

Blood samples (plasma [K_2_EDTA and heparin] and serum; BD vacutainers, Franklin Lakes, NJ) from unfasted pigs were obtained via jugular venipuncture prior to euthanasia. Plasma and serum samples were harvested by centrifugation at 4 °C and 1,500 × *g*, aliquoted, and stored at −80 °C until analysis. A second set of blood samples obtained in a K_2_EDTA tube were submitted to the Iowa State Department of Veterinary Pathology (Ames, IA) for automated-differential complete blood count analysis using a flow cytometry-based hematology analyzer (ADVIA 2120i; Siemens, Munich, Germany). Blood samples obtained in the heparin vacutainers were immediately analyzed (within 5 min) for blood chemistry, electrolytes, and blood gases using an i-STAT handheld blood analyzer and test cartridges (CG8^+^; Abbott Point of Care, Princeton, NJ).

For cytokine analysis, serum samples were submitted to the University of Minnesota Cytokine Reference Laboratory. Samples were analyzed with a porcine cytokine/chemokine immunology multiplex assay (PCYTMG-23K-13PX; MilliporeSigma; Burlington, MA) using antibodies to porcine granulocyte macrophage colony-stimulating factor (**GMCSF**), tumor necrosis factor alpha (**TNFα**), interleukin (**IL**) 1-β, IL-6, and IL-10. Samples were assayed according to manufacturer’s instructions on a MAGPIX Multiplex reader (Bio-Rad Laboratories, Hercules, CA) and analyzed with Belysa software (MiliporeSigma). All values that fell within the standard curve range had a coefficient of variation of less than 10%. The GMCSF and TNFα concentrations were below the detectable limits and were not considered for further analysis. Circulating non-esterified fatty acids (**NEFA**), histamine, cortisol, and CRF concentrations were determined using commercially available kits (NEFA, Wako Chemicals USA, Inc., Richmond, VA; histamine, Oxford Biomedical Research, Oxford, MI; cortisol, Enzo Life Sciences Inc., Farmingdale, NY; CRF, Biomatik, Wilmington, DE). The intra- and inter-assay coefficients of variations for NEFA, histamine, and cortisol were 3.0% and 9.1%, 4.7% and 8.9%, and 7.4% and 11.9%, respectively. The intra-assay coefficient of variation for CRF was 1.42%.

### Organ weights and tissue collection

Pigs were euthanized at the end of their respective environmental challenge with the captive bolt technique followed by exsanguination. Sections from the small and large intestines were immediately harvested. A jejunum sample was obtained approximately 90 cm distal to the pyloric sphincter, and an ileum sample was collected approximately 15 cm proximal to the ileocecal junction. A colon sample was obtained approximately 30 cm proximal to the rectum. Intestinal segments from the jejunum, ileum, and colon were flushed with cold 0.9% sodium chloride solution to remove remnant luminal contents. Then, stomach and small and large intestines were removed, and their contents were weighed. Weights of the empty stomach and small and large intestines were also recorded to determine total gastrointestinal tract weight.

### Histological analysis

For histological analysis, transversal sections were collected from each intestinal segment, fixed in 10% neutral buffered formalin for 24 h, and then transferred into 70% ethanol. Samples were submitted to the Iowa State Veterinary Comparative Pathology Core for sectioning and staining. May Grunwald Giemsa staining was used for mast cell quantification. Myeloperoxidase (**MPO**) immunohistochemistry staining was used as a marker of neutrophil infiltration. For each staining, one slide per pig per intestinal segment was generated (Supplementary Figure S1A and B). Using a microscope (Leica DMI3000 B Inverted Microscope, Bannockburn, IL) with an attached camera (QImaging 12-bit QICAM Fast 1394, Surrey, BC, Canada), five images per intestinal segment were obtained at ×400 magnification with the Q capture Pro software (QImaging). Image processing and quantification were completed using ImageJ software (U. S. National Institutes of Health, Bethesda, MD). For each intestinal segment, five non-overlapping areas above the muscularis mucosae were measured and later averaged to make *n* = 1. Mast cells were quantified using previously described methods (Dubuc et al., 2020) and expressed as cells/mm^2^ of tissue area. MPO was expressed as a percentage of positive MPO relative to the total stained area.

### Statistical analysis

Data were statistically analyzed using the MIXED procedure of SAS version 9.4 (SAS Inst. Inc. Cary, NC). The model included treatment as fixed effect, and initial BW was used as a covariate for all analyses. A logarithmic transformation was performed for cytokine data and results were back-transformed for interpretation. Pre-planned contrasts were assessed using the CONTRAST procedure in SAS to evaluate environmental (i.e., TNCon vs. HSCon and TNCon vs. HSKet) and therapeutic (i.e., HSCon vs. HSKet) effects. Pig was the experimental unit for all analyses. Results are reported as least squares means and considered significant when *P* ≤ 0.05 and tendency if 0.05 < *P* ≤ 0.10.

## Results

### Phenotypic responses to HS

Pigs exposed to HS had an overall increase in T_R_, T_S_, and RR compared to their TNCon counterparts (1.9 °C, 6.9 °C, and 119 bpm; *P* < 0.01; Table 2). Regardless of therapeutic treatment, pigs exposed to 12 h HS had increased T_R_ relative to their 2 and 6 h HS counterparts (0.5 °C; *P* < 0.05; Table 2). Furthermore, increased RR was observed in HSKet relative to HSCon pigs; however, this difference was mainly driven by increased RR in the 2 and 6 h HSKet pigs (+35 bpm; *P* < 0.01; Table 2).

**Table 2.** Effects of Ket on body temperature indices and gastrointestinal measurements during 2, 6, and 12 h acute HS exposure in growing pigs

Parameter	Treatment^1^							SEM	*P*-value	Contrasts^3^		
	TNCon	2 h		6 h		12 h			Trt^2^	TNCon vs. HSCon	TNCon vs. HSKet	HSCon vs. HSKet
		HSCon	HSKet	HSCon	HSKet	HSCon	HSKet					
Body temperature												
T_R_^4^, °C	39.0^c^	40.6^b^	40.8^b^	40.6^b^	40.8^b^	41.1^a^	41.2^a^	0.1	<0.01	<0.01	<0.01	0.13
T_S_^5^, °C	35.5^b^	43.0^a^	42.3^a^	41.9^a^	41.8^a^	42.3^a^	43.3^a^	0.6	<0.01	<0.01	<0.01	0.87
RR^6^, bpm	42^d^	159^bc^	215^a^	126^c^	174^b^	147^bc^	146^bc^	12	<0.01	<0.01	<0.01	<0.01
GIT Weight^7^, kg	7.44^a^	5.75^b^	5.50^b^	5.60^b^	5.36^b^	5.60^b^	5.35^b^	0.27	<0.01	<0.01	<0.01	0.27
Luminal contents, kg	4.27^a^	2.54^b^	2.50^b^	2.69^b^	2.33^b^	2.66^b^	2.49^b^	0.22	<0.01	<0.01	<0.01	0.30
Stomach, kg	1.96^a^	0.89^b^	0.93^b^	0.94^b^	0.73^b^	1.05^b^	0.99^b^	0.15	<0.01	<0.01	<0.01	0.52
Small intestine, kg	0.89^a^	0.41^b^	0.47^b^	0.60^b^	0.53^b^	0.55^b^	0.47^b^	0.07	<0.01	<0.01	<0.01	0.56
Large intestine, kg	1.43^x^	1.23^xy^	1.11^y^	1.15^y^	1.07^y^	1.06^y^	1.04^y^	0.09	0.07	0.01	<0.01	0.32
Empty GIT^8^, kg	3.17	3.21	3.00	2.91	3.03	2.94	2.86	0.10	0.13	0.23	0.10	0.46
Stomach, kg	0.41	0.45	0.40	0.41	0.41	0.40	0.41	0.01	0.30	0.83	0.66	0.32
Small intestine, kg	1.68	1.67	1.61	1.52	1.61	1.62	1.51	0.07	0.48	0.34	0.20	0.60
Large intestine, kg	1.08	1.10	1.00	0.98	1.01	0.92	0.93	0.05	0.16	0.24	0.14	0.66
Empty GIT^8^, % BW^9^	6.13	6.30	5.93	5.74	6.02	5.86	5.80	0.18	0.37	0.47	0.35	0.75
Stomach, % BW	0.80	0.88	0.78	0.80	0.81	0.80	0.83	0.03	0.17	0.52	0.83	0.50
Small intestine, % BW	3.26	3.27	3.17	3.01	3.20	3.22	3.07	0.12	0.69	0.55	0.46	0.82
Large intestine, % BW	2.07	2.15	1.98	1.94	2.01	1.84	1.89	0.10	0.41	0.43	0.38	0.88

^1^TNCon, thermoneutral control; HSCon, heat stress control; HSKet, heat stress ketotifen; 2-, 6-, and 12 h of HS exposure.

^2^Treatment.

^3^Contrasts: TNCon vs. HSCon (2 + 6 + 12-h HSCon); TNCon vs. HSKet (2 + 6 + 12-h HSKet); HSCon (2 + 6 + 12-h HSCon) vs. HSKet (2 + 6 + 12-h HSKet).

^4^Rectal temperature.

^5^Skin temperature.

^6^Respiration rate.

^7^Total gastrointestinal tract weight (luminal contents + empty gastrointestinal tract weight).

^8^Empty gastrointestinal tract.

^9^Empty gastrointestinal tract expressed as percentage of final BW.

^a-d^Means within the same row significantly differ (*P* ≤ 0.05).

^x,y^Means within the same row tend to differ (0.05 < *P* ≤ 0.10).

**Figure 1.**
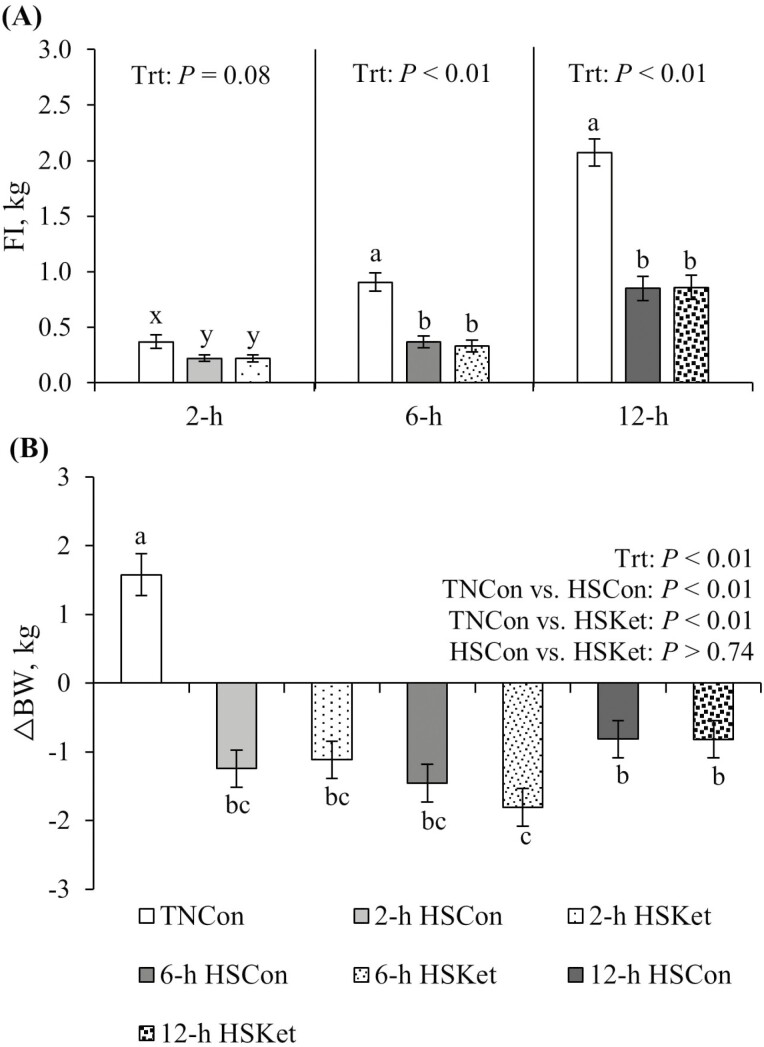
Effects of Ket during a 2-, 6-, and 12-h acute HS challenge in growing pigs. Treatments: TNCon, thermoneutral control; HSCon, heat stress control; HSKet, heat stress ketotifen. (A) FI was analyzed separately for each time point at 2 h (TNCon, *n* = 6; HSCon, *n* = 18; HSKet, *n* = 18), 6 h (TNCon, *n* = 6; HSCon, *n* = 12; HSKet, *n* = 12), and 12 h (TNCon, *n* = 6; HSCon, *n* = 6; HSKet, *n* = 6). (B) Delta BW was calculated as final − initial BW relative to the start of the environmental challenge. Data are represented as least squares means ± SE of the mean. ^a-c^Values with differing superscripts denote overall treatment differences (*P* ≤ 0.05). ^x,y^Values with differing superscripts denote tendencies (0.05 < *P* ≤ 0.10).

As expected, FI decreased in HS pigs at 2, 6, and 12 h (40%, 61%, and 59%, respectively; *P* ≤ 0.08; Figure 1A) relative to their TNCon counterparts. Irrespective of therapeutic treatment, FI slightly increased over time from 2 to 12 h of HS exposure (0.22, 0.35, and 0.85 kg, respectively; *P* < 0.01). Overall, a 59% reduction in FI was observed in the 12 h HS pigs relative to their TNCon counterparts (0.85 vs. 2.07 kg; *P* < 0.01; Figure 1A). In addition, pigs exposed to HS lost BW, while TNCon pigs gained BW (−1.21 vs. +1.51 kg; *P* < 0.01; Figure 1B).

### Gastrointestinal measurements

Total gastrointestinal tract weight (luminal contents + empty tissue) was reduced in HS pigs compared to their TNCon counterparts (28%; *P* < 0.01; Table 2). This reduction was mainly associated with decreased luminal contents along the alimentary tract as empty gastrointestinal tissue weight (absolute and as a percentage of BW) was not altered during HS (*P* > 0.13; Table 2). Regardless of therapeutic treatment, stomach and small intestinal contents decreased similarly from 2 to 12 h of HS exposure, relative to TNCon pigs (0.95 vs. 1.96 kg and 0.51 vs. 0.89 kg, respectively; *P *< 0.01; Table 2). Likewise, large intestinal contents tended to decrease in HS relative to TNCon pigs (1.11 vs. 1.43 kg; *P* = 0.07; Table 2).

### Circulating CRF, cortisol, and histamine

**Figure 2.**
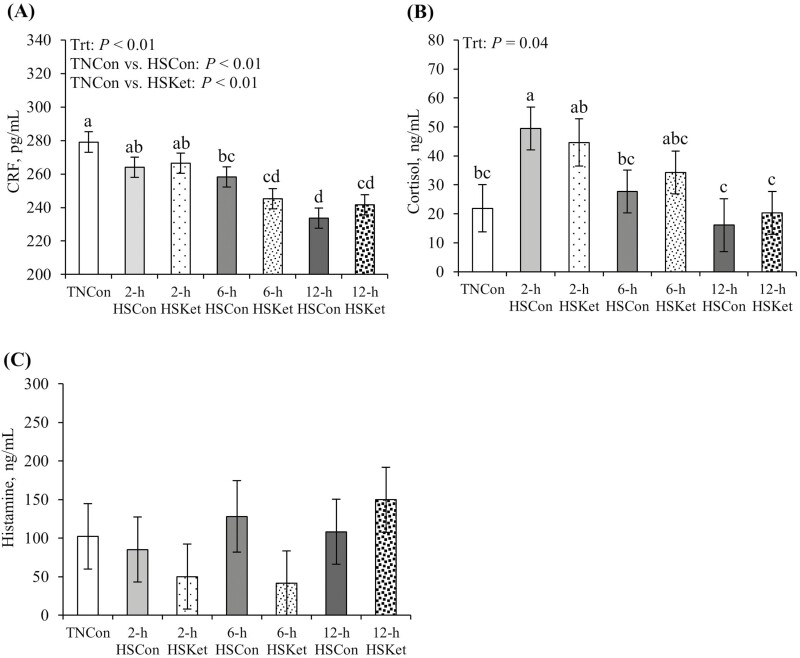
Effects of Ket on circulating (A) CRF, (B) cortisol, and (C) histamine during a 2-, 6-, and 12-h acute HS challenge in growing pigs. Treatments: TNCon, thermoneutral control; HSCon, heat stress control; HSKet, heat stress ketotifen. Data are represented as least squares means ± SE of the mean. ^a-d^Values with differing superscripts denote overall treatment differences (*P* ≤ 0.05).

Regardless of therapeutic treatment, plasma CRF consistently decreased from 2 to 12 h of HS exposure relative to TNCon pigs (*P* < 0.01; Figure 2A). Compared to TNCon pigs, circulating cortisol increased at 2 h of HS exposure (2-fold); subsequently, cortisol gradually returned to TN levels by 6 h of HS and continued to decrease by 12 h (*P* = 0.04; Figure 2B). No differences in circulating cortisol levels were detected between Con and Ket pigs (*P* > 0.75). Plasma histamine concentrations did not differ between environmental or therapeutic treatments (*P* > 0.54; Figure 2C).

### Intestinal mast cells and MPO

Regardless of therapeutic treatment and time of HS exposure, jejunum mast cells increased in HS relative to TN controls (95%; *P* < 0.01; Table 3). Ileum mast cell numbers remained similar among treatments (*P* > 0.51). However, regardless of therapeutic treatment, post hoc analysis indicates a tendency for increased (30%) mast cell numbers at 12 h after HS initiation compared to TN controls (*P* = 0.10; data not shown). Overall, no environmental or therapeutic effects were detected on colon mast cell numbers (*P* > 0.21; Table 3); however, a post hoc analysis indicates a tendency for reduced (43%) mast cells in 12 h HSKet relative to 12 HSCon pigs (*P* = 0.10; data not shown). MPO area remained similar across treatments in the jejunum, ileum, and colon (*P* > 0.58; Table 3). However, a tendency for increased colon MPO area was observed in HSCon pigs relative to their TNCon counterparts (12%; *P* = 0.08; Table 3).

**Table 3.** Effects of Ket on intestinal MPO and mast cell counts during 2, 6, and 12 h acute HS exposure in growing pigs

Parameter	Treatment							SEM	*P*-value	Contrasts^1^		
	TNCon	2 h		6 h		12 h			Trt	TNCon vs. HSCon	TNCon vs. HSKet	HSCon vs. HSKet
		HSCon	HSKet	HSCon	HSKet	HSCon	HSKet					
Mast cells^2^, cells/mm^2^												
Jejunum	158^y^	302^x^	314^x^	284^x^	290^x^	345^x^	314^x^	38	0.06	<0.01	<0.01	0.89
Ileum	650	743	667	716	678	813	875	88	0.51	0.33	0.39	0.82
Colon	116	82	97	88	126	105	60	18	0.21	0.30	0.32	0.89
MPO area^3^, %												
Jejunum	1.50	1.51	1.47	1.54	1.57	1.51	1.46	0.05	0.65	0.64	0.94	0.59
Ileum	2.43	2.39	2.35	2.40	2.49	2.49	2.32	0.09	0.80	0.97	0.68	0.60
Colon	0.85	0.96	0.88	0.92	0.89	0.97	0.93	0.05	0.58	0.08	0.37	0.22

^1^Contrasts: TNCon vs. HSCon (2 + 6 + 12-h HSCon); TNCon vs. HSKet (2 + 6 + 12-h HSKet); HSCon (2 + 6 + 12-h HSCon) vs. HSKet (2 + 6 + 12-h HSKet).

^2^Expressed as cells/mm^2^ of tissue area.

^3^Expressed as a percentage of positive MPO relative to the total stained area.

^x,y^Means within the same row tend to differ (0.05 < *P* ≤ 0.10).

TNCon, thermoneutral control; HSCon, heat stress control; HSKet, heat stress ketotifen; 2, 6, and 12 h of HS exposure; Trt, treatment.

### Hematology parameters and circulating cytokines

**Figure 3.**
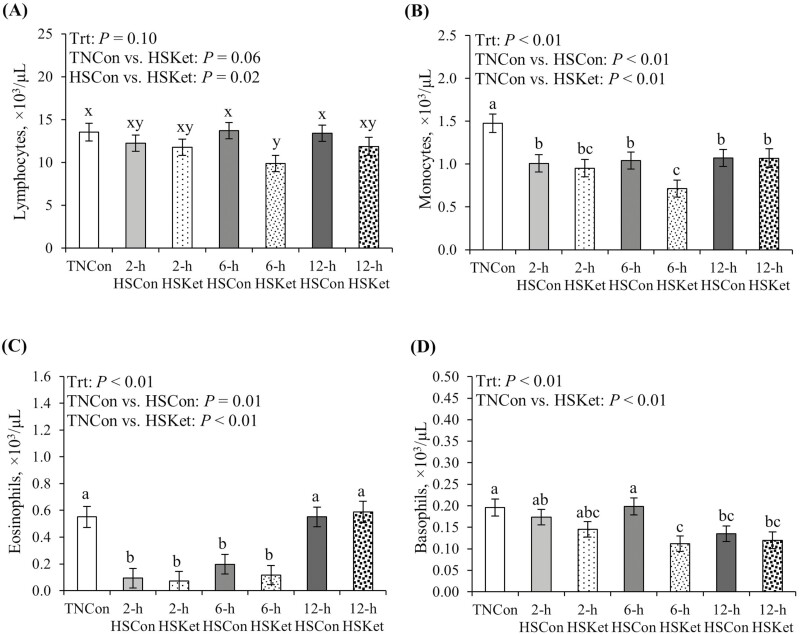
Effects of Ket on circulating (A) lymphocytes, (B) monocytes, (C) eosinophils, and (D) basophils during a 2-, 6-, and 12-h acute HS challenge in growing pigs. Treatments: TNCon, thermoneutral control; HSCon, heat stress control; HSKet, heat stress ketotifen. Data are represented as least squares means ± SE of the mean. ^a-c^Values with differing superscripts denote overall treatment differences (*P* ≤ 0.05). ^x,y^Values with differing superscripts denote tendencies (0.05 < *P* ≤ 0.10).

No overall differences in circulating white blood cells were observed among treatments (*P* > 0.15; Table 4); however, across time points, HSKet pigs had reduced leukocyte counts relative to TNCon pigs (*P* = 0.04; Table 4). Circulating neutrophils were similar across treatments (*P* > 0.84). However, lymphocytes tended to decrease and were decreased in HSKet pigs relative to TN and HSCon pigs (17% and 15%, respectively; *P* ≤ 0.06; Figure 3A). Blood monocytes and eosinophils were reduced in HS relative to TNCon pigs (34% and 51%, respectively; *P* < 0.01; Figure 3B and C). Furthermore, circulating basophils decreased over time in HSKet pigs relative to TNCon and HSCon pigs (37% and 25%, respectively; *P* ≤ 0.01 Figure 3D). No other differences in red blood cells, hemoglobin, hematocrit, or platelets were observed among treatments (*P* > 0.71; Table 4).

**Table 4.** Effects of Ket on hematology parameters and circulating cytokines during 2, 6, and 12 h acute HS exposure in growing pigs

Parameter	Treatment							SEM	*P*-value	Contrasts^1^		
	TNCon	2 h		6 h		12 h			Trt	TNCon vs. HSCon	TNCon vs. HSKet	HSCon vs. HSKet
		HSCon	HSKet	HSCon	HSKet	HSCon	HSKet					
Hematology												
WBC, ×10^3^/μL	25.5	23.4	21.8	24.7	20.0	23.1	23.7	1.4	0.15	0.29	0.04	0.11
Neut., ×10^3^/μL	9.60	9.76	8.69	9.33	9.07	7.78	9.83	1.10	0.84	0.63	0.77	0.77
RBC, ×10^3^/μL	6.83	6.92	6.74	6.99	6.61	6.99	6.82	0.20	0.71	0.53	0.63	0.10
Hemoglobin, g/dL	12.0	12.0	11.8	11.8	11.6	12.4	12.0	0.3	0.47	0.71	0.59	0.20
Hematocrit, %	35.2	35.3	34.7	34.7	34.0	36.5	35.3	0.8	0.46	0.74	0.57	0.20
Cytokines												
IL-1β, pg/mL^2^	7.94	2.26	3.96	3.26	1.32	2.98	4.77	0.70	0.14	0.06	0.07	0.89
IL-6, pg/mL^3^	12.08	1.55	6.08	3.23	2.04	3.96	6.01	0.86	0.11	0.02	0.09	0.27
IL-10, pg/mL^2^	29.97	10.13	13.23	7.29	5.71	10.26	49.23	5.66	0.37	0.20	0.48	0.35

^1^Contrasts: TNCon vs. HSCon (2 + 6 + 12-h HSCon); TNCon vs. HSKet (2 + 6 + 12-h HSKet); HSCon (2 + 6 + 12-h HSCon) vs. HSKet (2 + 6 + 12-h HSKet).

^2^Values were log-transformed using log base 10 (log_10_) and back-transformed for interpretation.

^3^Values were log-transformed using log base 10 + 1 (log_10_ [1 + *x*]) and back-transformed for interpretation.

TNCon, thermoneutral control; HSCon, heat stress control; HSKet, heat stress ketotifen; 2, 6, and 12 h of HS exposure; Trt, treatment; WBC, white blood cells; Neut., neutrophils; RBC, red blood cells; IL-1β, interleukin-1β; IL-6, interleukin-6; IL-10, interleukin-10.

Overall, no differences in circulating IL-1β, IL-6, and IL-10 were observed across treatments (*P* > 0.11; Table 4). However, irrespective of time, IL-1β tended to be decreased in HSCon (64%; *P* = 0.06) and HSKet pigs (57%; *P* = 0.07) relative to TN controls. Similarly, IL-6 was decreased in HSCon (63%; *P* = 0.02) and tended to be decreased in HSKet pigs (41%; *P* = 0.09), when compared to their TNCon counterparts. No therapeutic effects of Ket were detected in any of the circulating cytokines measured herein.

### Blood chemistry, electrolytes, and blood gases

Blood glucose, Na, and K concentrations did not differ among treatments (*P *> 0.20; Table 5). Plasma NEFA concentrations were increased in HSKet relative to TN and HSCon pigs (2-fold; *P* = 0.02); however, this effect was more evident at 6 and 12 h of HS exposure (Table 5). Blood ionized Ca (**iCa**) decreased in HS pigs relative to their TNCon counterparts (1.24 vs. 1.33 mmol/L; *P* < 0.01), and the nadir occurred at 6 h of HS exposure (Table 5).

**Table 5.** Effects of Ket on blood chemistry, electrolytes, hematology, and blood gases during 2, 6, and 12 h acute HS exposure in growing pigs

Parameter	Treatment							SEM	*P*-value	Contrasts^1^		
	TNCon	2 h		6 h		12 h			Trt	TNCon vs. HSCon	TNCon vs. HSKet	HSCon vs. HSKet
		HSCon	HSKet	HSCon	HSKet	HSCon	HSKet					
Chemistry/electrolytes												
Glucose, mg/dL	113	108	113	109	105	108	103	4	0.67	0.37	0.24	0.69
NEFA, mEq/L	106^c^	132^c^	111^c^	158^bc^	279^a^	123^c^	254^ab^	39	0.01	0.49	0.02	0.02
iCa, mmol/L	1.33^a^	1.29^a^	1.22^bc^	1.22^bc^	1.19^c^	1.26^ab^	1.26^ab^	0.02	<0.01	0.01	<0.01	0.12
Na, mmol/L	140	142	142	141	142	144	142	1	0.31	0.09	0.16	0.63
K, mmol/L	5.06	4.71	4.46	5.17	4.93	5.11	4.75	0.21	0.20	0.80	0.16	0.10
Blood gases												
pH	7.34^b^	7.43^a^	7.44^a^	7.37^b^	7.43^a^	7.37^b^	7.44^a^	0.02	<0.01	0.02	<0.01	<0.01
pCO_2_, mmHg	52.9^a^	42.7^b^	39.7^b^	46.2^b^	43.2^b^	45.6^b^	42.1^b^	2.4	0.01	0.01	<0.01	0.11
pO_2_, mmHg	31.4	39.1	46.2	39.2	31.5	28.2	33.2	5.6	0.30	0.54	0.39	0.75
TCO_2_, mmol/L	30.8	29.5	28.1	28.0	29.6	27.6	30.1	1.5	0.66	0.16	0.37	0.44
HCO_3_, mmol/L	29.1	28.2	27.0	26.7	28.4	26.2	28.8	1.5	0.72	0.23	0.56	0.37
Beecf, mmol/L	3.35	4.02	2.79	1.33	4.08	0.79	4.81	1.55	0.49	0.47	0.76	0.15
sO_2_, %	54.2	67.5	77.2	65.8	59.1	50.0	57.4	7.7	0.23	0.45	0.26	0.59

^1^Contrasts = TNCon vs. HSCon (2 + 6 + 12-h HSCon); TNCon vs. HSKet (2 + 6 + 12-h HSKet); HSCon (2 + 6 + 12-h HSCon) vs. HSKet (2 + 6 + 12-h HSKet).

^a-c^Means within the same row significantly differ (*P* ≤ 0.05).

TNCon, thermoneutral control; HSCon, heat stress control; HSKet, heat stress ketotifen; 2, 6, and 12 h of HS exposure; Trt, treatment; NEFA, non-esterified fatty acids; iCa, ionized calcium; pCO_2_, partial pressure of carbon dioxide; pO_2_, partial pressure of oxygen; TCO_2_, total carbon dioxide; HCO_3_, bicarbonate; Beecf, base excess in the extracellular fluid compartment; sO_2_, oxygen saturation.

Blood pH increased at 2 h of HS exposure, relative to TNCon pigs (*P* < 0.01). Subsequently, blood pH returned to TN levels in HSCon pigs but remained elevated in HSKet pigs (*P* < 0.01; Table 5). In addition, pCO_2_ decreased similarly in HSCon and HSKet from 2 to 12 h of HS exposure, compared to TNCon pigs (*P* = 0.01; Table 5). No other differences in blood gases were observed due to environmental or therapeutic treatment (*P* > 0.23; Table 5).

## Discussion

HS is one of the costliest issues in animal agriculture as it undermines animal welfare and limits growth efficiency and productivity (St-Pierre et al., 2003; Key and Sneeringer, 2014). Although mechanisms responsible for decreased animal productivity during HS are not fully understood, they appear to originate from the gastrointestinal tract (Pearce et al., 2013; Koch et al., 2019; Nanto-Hara et al., 2020). HS compromises intestinal barrier integrity, allowing bacterial antigens (i.e., lipopolysaccharide [**LPS**]) to translocate across the gut wall and enter circulation (Hall et al., 2001; Lambert et al., 2002; Pearce et al., 2014). The resultant immune activation triggers local and systemic inflammatory responses that are nutrient and energetically demanding (Kvidera et al., 2017; Huntley et al., 2018).

A potential mechanism by which environmental hyperthermia induces gut barrier dysfunction involves the stress response to the heat load. Recent evidence indicates that stress alone induces intestinal barrier dysfunction, leading to increased gut permeability and inflammation, a process likely mediated by the CRF signaling pathway (Santos et al., 2001; Wallon et al., 2008; Overman et al., 2012). The connection between CRF and intestinal barrier dysfunction appears to be mediated by mast cells, which, upon activation, degranulate and release a wide array of compounds that can negatively influence intestinal barrier integrity (Santos et al., 2001). Mast cell activation and degranulation play an important role in the pathogenesis of intestinal dysfunction in various inflammatory disorders (Wouters et al., 2016) and other seemingly unrelated stressors (Moeser et al., 2007a, b; Li et al., 2017); however, their contribution to intestinal barrier permeability during HS is poorly understood. Therefore, characterizing mast cells’ role during a thermal load is foundational to better understand the etiology of HS-induced intestinal dysfunction and for developing ameliorative strategies. Thus, the central aim of our study was to investigate the temporal pattern of intestinal mast cell dynamics during an acute HS event in growing pigs and to evaluate the therapeutic effects of mast cell stabilization.

By design, the current HS protocol induced a significant increase in all body thermal indices, including T_R_, T_S_, and RR, a response comparable in magnitude to that reported in our previous acute HS studies (Mayorga et al., 2020, 2021). In addition, pigs exposed to 12-h HS had increased T_R_ relative to the 2-h and 6-h HS treatments, suggesting pigs accumulated more heat over time as the exposure to the thermal load progressed. Further, Ket-treated pigs had increased RR at 2 and 6 h after HS initiation compared to their HSCon counterparts; however, the biological relevance of this observation remains unknown.

Severe hypophagia was also observed in pigs exposed to the acute heat load. Although FI slightly increased over time during HS, it remained below that observed in TNCon pigs, and by 12 h of HS exposure, the reduction in FI was nearly 60% compared to their TNCon counterparts. This drastic reduction in FI is characteristic of animals exposed to HS and represents a survival strategy to reduce metabolic heat production associated with the digestion and absorption of nutrients (Collin et al., 2001). Along with reduced FI, pigs exposed to HS lost BW relative to their TNCon counterparts, and this response occurred as early as 2 h after HS initiation. We and others have previously reported that pigs exposed to an acute HS challenge have a marked reduction in BW (Pearce et al., 2014; Mayorga et al., 2020, 2021). In those studies, BW loss was only partially explained by the decrease in FI, suggesting mechanisms other than reduced FI influence this response. Thus, understanding how an acute thermal event negatively influences FI and BW is important to understanding how HS affects bioenergetics and nutrient partitioning.

Changes in carbohydrate metabolism are characteristic of animals exposed to HS, and evidence suggests an increased reliance on glucose as a substrate during hyperthermia (Febbraio, 2001; Garriga et al., 2006; Baumgard et al., 2011), which is often accompanied by hypoglycemia (Wheelock et al., 2010; Sanz Fernandez et al., 2015). However, in the current study, circulating glucose remained similar between environmental and therapeutic treatments. Changes in circulating glucose during HS are not always consistent, as some have reported increased (Prunier et al., 1997), decreased (Sanz Fernandez et al., 2015; Abuajamieh et al., 2018), or no differences (Mayorga et al., 2021) in response to a heat load. Thus, considering the tight regulation of glucose homeostasis, the lack of effects in this metabolite may be influenced by the timing of sampling relative to the onset of HS.

HS attenuates lipid mobilization, an effect that is more apparent when comparing TN and HS animals under the same plane of nutrition (Pearce et al., 2013; Sanz Fernandez et al., 2015). Herein, reduced lipid mobilization was only evident in HSCon pigs, whereas HSKet animals had increased circulating NEFA at 6 and 12 h of HS exposure. It is unclear why administering Ket would increase lipid mobilization during HS, particularly when no apparent differences exist between Con and Ket-treated pigs in other phenotypical responses like FI or BW. Nevertheless, it is worth emphasizing that the extent of lipid mobilization observed in HSKet pigs is still small compared to that reported in underfed or malnourished animals (Govoni et al., 2005; Metges et al., 2015).

Together with changes in metabolism, we evaluated the stress response to the heat load and its consequences on mast cell activation and degranulation. Surprisingly, circulating CRF decreased progressively over time. The mechanisms behind this response are not entirely clear, but possible explanations include a negative feedback loop from increased cortisol (Gjerstad et al., 2018), elevated CRF binding protein (Behan et al., 1995), or enhanced leukocyte extravasation into tissues and thus less circulating white blood cells actually secreting CRF (Smith, 2007). The latter premise is supported by the temporal pattern of circulating leukocytes observed herein, as described later. Further, we observed a marked increase in circulating cortisol that was evident 2 h after HS initiation, after which it decreased to basal levels. This gradual decrease in circulating cortisol could be attributed to cortisol’s ability to regulate its own synthesis by exerting negative feedback at the hypothalamus and the pituitary gland, thus inhibiting the release of CRF and ACTH, respectively (Gjerstad et al., 2018).

Increased circulating cortisol has been previously reported in various species in response to acute and chronic HS (Wang et al., 2015; Chen et al., 2018; Al-Qaisi et al., 2020). The initial increase in plasma cortisol observed herein suggests HS induced a rapid stress stimulus that triggered the activation of the HPA axis early during the thermal load. Because mast cells are considered important effectors in the HPA axis, their activation, and degranulation in response to stress may have resulted in the rapid release of pre-formed mediators, such as histamine, which negatively influences intestinal barrier integrity and function (Potts et al., 2016). However, contrary to expectations, circulating histamine concentrations were not affected by HS or length of thermal exposure. How HS affects circulating histamine is unknown, but increased levels have been reported in humans after heat exposure and physical exercise (Garden, 1966). Reasons for the absence of a histamine response in the current study might indicate a lack of mast cell activation during HS; however, we did not measure other markers of mast cell degranulation (e.g., tryptase) to support this premise. Alternatively, the lack of histamine effects may be explained by its transient pattern in circulation, as it can be spontaneously released upon mast cell degranulation and return to normal levels within 60 min after the onset of the stress event (Lieberman and Ewan, 2012). Presumably, histamine content in the intestinal mucosa is more reflective of the degree of mast cell degranulation than histamine concentrations in systemic circulation (Raithel et al., 1995). However, whether this limited our ability to detect histamine differences in our current study remains unknown. Thus, the relevance of histamine as an indicator of mast cell activation during HS merits additional investigation.

Compromised intestinal barrier integrity and function occur in response to HS in various species (Pearce et al., 2013, 2014; Koch et al., 2019; Nanto-Hara et al., 2020). Depending on the severity and length of the HS episode, the magnitude of these alterations can occur within minutes or hours of HS exposure. Accordingly, in a rodent model of acute HS, intestinal epithelial sloughing occurred as early as 30 min, and intestinal permeability was apparent after 60 min (Lambert et al., 2002). Similarly, changes in intestinal architecture and permeability have been observed in pigs as early as 2 h after HS initiation, and these alterations remained apparent even after 12 h of HS exposure (Pearce et al., 2014, 2015). Thus, to determine the extent to which mast cells contribute to altered intestinal integrity and inflammation during a heat load, a temporal pattern of HS was evaluated. Herein, increased jejunum mast cell numbers were observed in HS pigs as early as 2 h of HS exposure, and numbers remained consistently elevated at 6 and 12 h. In addition, HS tended to increase ileum mast cell numbers (30%) after 12 h of HS, but no differences in mast cell abundance were detected in the colon. Increased mast cell numbers could be interpreted as increased mast cell recruitment and or mast cell precursor maturation within intestinal tissue in response to an inflammatory stimulus (Halova et al., 2012). However, whether this was translated into mast cell activation remains unknown, especially considering the lack of a histamine response (as previously discussed). Mast cells’ role during HS is currently ill-defined; however, increased mast cell numbers and mast cell activation have been repeatedly shown to play a crucial role in the onset of intestinal barrier dysfunction in various stress-related gastrointestinal disorders, including irritable bowel syndrome, inflammatory bowel diseases, and chronic diarrhea (Farhadi et al., 2007; Smith et al., 2010; Pohl et al., 2017). Thus, while our observations support the premise that mast cells are key mediators in the etiology of HS-induced intestinal hyperpermeability, further research is needed to understand the mechanisms underlying this response.

Besides mast cell quantification, intestinal MPO area was assessed as a marker of neutrophil infiltration. Neutrophils are recruited from circulation in response to local infection or inflammatory signals, a process likely mediated by tissue-resident leukocytes such as mast cells and macrophages (De Filippo et al., 2013). Herein, we observed a moderate increase (12%) in colon MPO area in HSCon pigs relative to TN controls, while no other environmental or therapeutic effects were detected in the jejunum or ileum. Although unexpected, the absence of intestinal MPO effects coincided with a similar response in systemic circulation, where no changes in neutrophil counts were detected due to environmental or therapeutic treatments.

Contrary to our expectations, Ket did not alter circulating histamine levels or intestinal mast cell numbers in the present study. This was unexpected, considering Ket’s role in modulating mast cell activation and preventing intestinal inflammation (Eliakim et al., 1992; Serna et al., 2006). In farm animals, the use of Ket for the treatment of intestinal inflammatory conditions is limited. However, similar stabilizing agents prevented mast cell activation, reduced intestinal barrier dysfunction, and improved growth in a pig model of weaning stress (Moeser et al., 2007b; Mereu et al., 2015). Although the absence of therapeutic effects observed herein is surprising, our results corroborate others where Ket administration did not impact mast cell infiltration, degranulation, or histamine tissue concentrations in different inflammatory conditions (Klooker et al., 2010; Burks et al., 2019). Further, Mereu et al. (2015) observed no differences in intestinal mast cell degranulation or mast cell-tryptase concentration in weaning pigs treated with a mast cell stabilizer agent despite its beneficial effects on intestinal integrity and growth performance. Perhaps, inconsistencies in the pharmacologic activity of Ket across studies result from species differences and dissimilar dose regimens. In addition, our interpretation is based on systemic circulation, and it is likely that more pronounced changes would be detected when evaluating immunological changes specifically within the splanchnic bed. Further, Ket also acts as a histamine receptor antagonist and inhibits the effects of various proinflammatory mediators (i.e., eicosanoids), suggesting it likely exerts beneficial effects through mechanisms that are not specific to reducing mast cell activation (Eliakim et al., 1992; Kalia et al., 2005; Klooker et al., 2010). To the authors’ knowledge, no previous literature evaluating Ket’s role during HS exists; thus, further investigating the benefits of this potential therapeutic approach has pragmatic implications for animal agriculture.

We also evaluated circulating white blood cells and cytokine dynamics to further assess the inflammatory response to the heat load. Herein, HSKet pigs had an overall reduction in circulating leukocytes relative to TNCon pigs, a decrease that was influenced by reduced lymphocyte and basophil counts in Ket-treated pigs relative to their TN and HSCon counterparts. Interestingly, the patterns of circulating lymphocytes, monocytes, and basophils in HSKet pigs reflected each other, and the lowest values were observed at 6 h after HS initiation. Reasons for this particular response in Ket-treated pigs are not entirely clear; however, it may be related to Ket’s anti-inflammatory activity as it has been shown to reduce cytokine and chemokine gene expression at the cell (monocytes) and tissue (spleen) levels (Hung et al., 2007; Ryang et al., 2007). In addition, and regardless of therapeutic treatment, the acute heat load induced a transient decrease in circulating monocytes and eosinophils. Interestingly, monocytes remained consistently decreased, even after 12 h of HS exposure, while eosinophils returned to TN levels by the end of the HS challenge. Albeit with some exceptions, changes in circulating leukocytes observed herein agree with previous HS studies (Ju et al., 2014; Mayorga et al., 2021). Presumably, the decrease in monocyte and eosinophil counts during HS can be related to a simultaneous increase in leukocyte migration into injured tissue in response to an inflammatory stimulus (Shi and Pamer, 2011). An example is the study reported by Koch et al. (2019) in which HS increased macrophage trafficking into the bovine intestinal mucosa, likely from recruited monocytes in circulation. In addition, eosinophil accumulation in the gastrointestinal tract has been reported in various inflammatory disorders (Loktionov, 2019), and intestinal eosinophils appear to be an important source of peripheral CRF (Zheng et al., 2009).

Systemic inflammation during HS can also result from increased proinflammatory cytokines (Leon, 2007). However, we detected an overall decrease in IL-1β and IL-6 in heat-stressed pigs. Although counterintuitive, we and others have previously reported decreased circulating cytokines in response to acute and chronic HS in pigs (Pearce et al., 2014, 2015; Gabler et al., 2018). Increased cortisol can be anti-inflammatory (Padgett and Glaser, 2003), and the temporal pattern of this glucocorticoid may help explain the effects herein. Additionally, reduced IL-1β and IL-6 may be mediated by activated heat shock proteins (**HSP**, not measured herein) in response to the thermal load. Particularly, HSP inhibits the expression of the nuclear factor kappa B (**NF-κB**, not measured herein) pathway and thus the synthesis of proinflammatory cytokines (Dokladny et al., 2010). In fact, systemic inflammatory responses appear to be exacerbated after the cessation of HS, likely due to reduced HSP activity following heat removal (Johnson et al., 2016; Abuajamieh et al., 2018).

Another characteristic response to immune activation is a marked reduction in circulating Ca levels. Hypocalcemia has been repeatedly observed during infection or following LPS administration in various species (Carlstedt et al., 2000; Horst et al., 2020). Although mechanisms behind this response remain poorly understood, changes in cellular Ca uptake or increased tissue sequestration likely explain the reduction in circulating Ca during inflammation (Carlstedt et al., 2000). Herein, we observed a transient decrease in blood iCa in response to the heat load, and the nadir occurred after 6 h of HS exposure. Our observation agrees with others where HS induced a comparable reduction in circulating Ca levels (Odom et al., 1986; Felver-Gant et al., 2014). In addition, acute HS increased Ca uptake by splenic lymphocytes, presumably as a mechanism to improve immune cell proliferation (Han et al., 2010). Calcium sequestration from circulation has been proposed as a protective strategy that facilitates LPS detoxification by lipoproteins (Skarnes and Chedid, 1964), likely contributing to the hypocalcemia observed herein. Due to Ca’s involvement in immune activation, investigating the role of hypocalcemia during HS is warranted.

## Conclusion

Our study confirmed the characteristic phenotypical and physiological alterations observed in previous acute HS studies in pigs, including a marked increase in all body temperature indices, drastic FI reduction, BW loss, and changes in gastrointestinal mass metrics. Acute HS triggered a rapid increase in circulating cortisol and increased jejunum mast cell numbers as early as 2 h after the onset of HS. Although Ket administration had little to no effects on markers of mast cell activation, it appeared to influence lipid metabolism and leukocyte dynamics. Furthermore, the current HS protocol induced transient changes in circulating leukocytes and iCa indicative of immune activation, presumably due to altered intestinal integrity and function. Whether these alterations result from stress-induced mast cell activation remains unclear but deserves further investigation.

## Supplementary Material

skae030_suppl_Supplementary_Figures_S1

## Abbreviations

bpm: breaths per minute
BW: body weight
Con: control
CRF: corticotropin-releasing factor
FI: feed intake
GMCSF: granulocyte macrophage colony-stimulating factor
HPA: hypothalamic-pituitary-adrenal axis
HS: heat stress
HSCon: heat stress control
HSKet: heat stress ketotifen
IL: interleukin
Ket: ketotifen
LPS: lipopolysaccharide
MPO: myeloperoxidase
NEFA: non-esterified fatty acids
P: period
RR: respiration rate
TN: thermoneutral
TNCon: thermoneutral control
TNFα: tumor necrosis factor alpha
T_R_: rectal temperature
T_S_: skin temperature
