# Supplementary material for: Investigating intestinal mast cell dynamics during acute heat stress in growing pigs
Source: J Anim Sci. 2024 Jan 30;102:skae030. doi: 10.1093/jas/skae030 (PMC10889722; doi:10.1093/jas/skae030)
Supplement: skae030_suppl_Supplementary_Figures_S1 [file skae030_suppl_supplementary_figures_s1.docx]

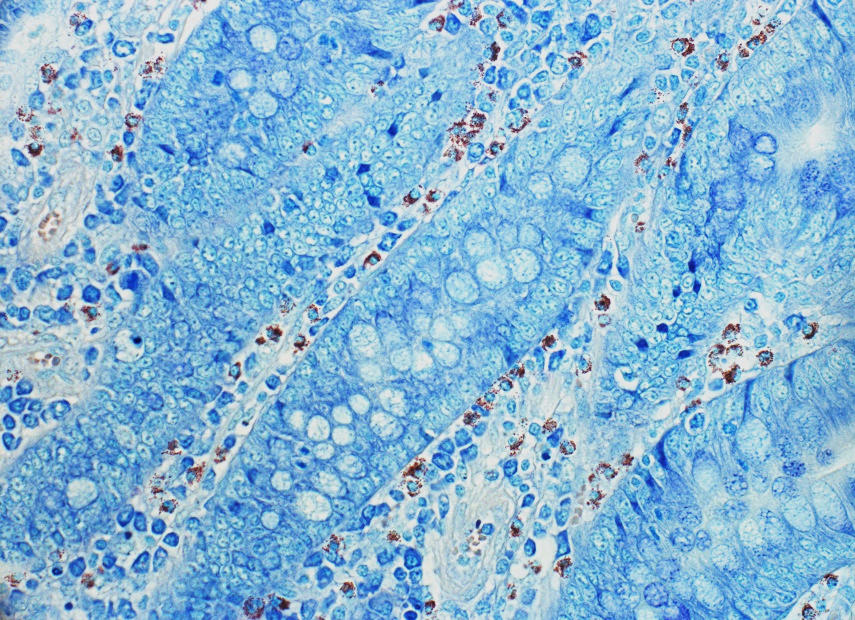


**100 µm**

**(A)**


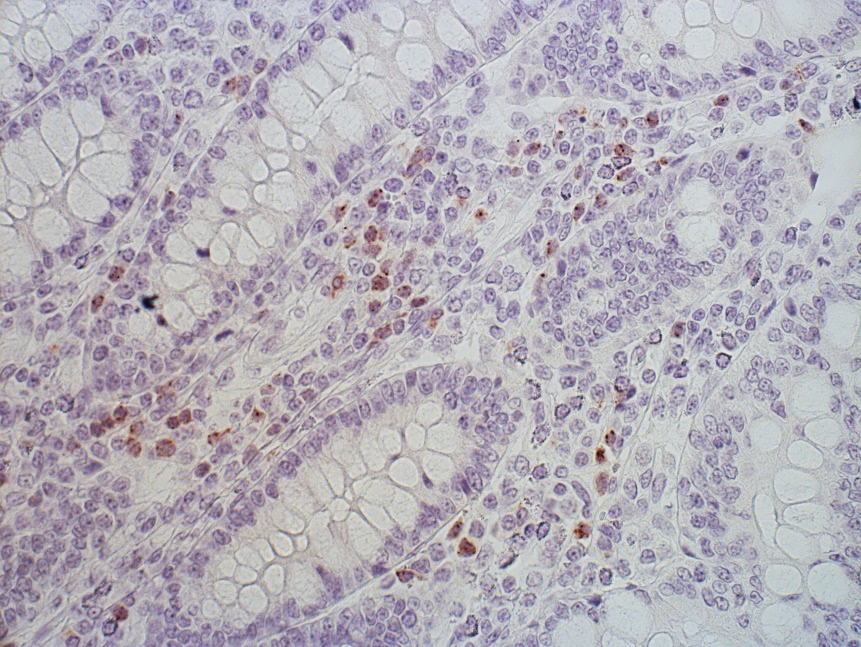


**(B)**

**100 µm**

**Supplementary Figure S1.** (A) Representative slide of an intestinal section stained with May Grunwald Giemsa for total mast cells quantification (original magnification 400×). (B) Representative slide of an intestinal section with positive immunohistochemical stain for myeloperoxidase (MPO) as a marker of neutrophil infiltration (original magnification 400×).
